# Identifying and articulating the student experience in the Intercalated Enrichment Year

**DOI:** 10.1186/s12909-022-03303-z

**Published:** 2022-04-04

**Authors:** Eric Yuk Fai Wan, Zhihao Li, Kai Sing Sun, Karina Hiu Yen Chan, Will Ho Gi Cheng, Julie Yun Chen, Weng Yee Chin, Tai Pong Lam, George Lim Tipoe, Gordon Tin Chun Wong, Sarah So Ching Chan, Cindy Lo Kuen Lam

**Affiliations:** 1 Department of Family Medicine and Primary Care, School of Clinical Medicine, Li Ka Shing Faculty of Medicine, The University of Hong Kong, 3/F Ap Lei Chau Clinic, 161 Main Street, Ap Lei Chau, Hong Kong SAR, China; 2 Department of Pharmacology and Pharmacy, Li Ka Shing Faculty of Medicine, The University of Hong Kong, Hong Kong SAR, China; 3 The Jockey Club School of Public Health and Primary Care, The Chinese University of Hong Kong, Hong Kong SAR, China; 4 Bau Institute of Medical and Health Sciences Education, Li Ka Shing Faculty of Medicine, The University of Hong Kong, Hong Kong SAR, China; 5 School of Biomedical Sciences, Li Ka Shing Faculty of Medicine, The University of Hong Kong, Hong Kong SAR, China; 6 Department of Anaesthesiology, School of Clinical Medicine, Li Ka Shing Faculty of Medicine, The University of Hong Kong, Hong Kong SAR, China

**Keywords:** Intercalation enrichment year, Undergraduate medicine curriculum, Learning experience, Mixed-method study

## Abstract

**Background:**

Benefits of intercalation during an undergraduate medical degree are well-recognized. The University of Hong Kong implemented a compulsory Enrichment Year (EY) in its Bachelor of Medicine and Bachelor of Surgery degree programme (MBBS) in 2016. In their third year of study, students could work on an area of interest in any of three programme categories (i) intercalation/ university exchange (IC); (ii) research (RA); (iii) service/ humanitarian work (SH). This study aimed to explore the barriers, enablers, and overall student learning experiences from the first cohort of EY students in order to inform future development of the EY.

**Methods:**

An exploratory sequential mixed-method study in 2019-20. Twenty students were purposively selected to attend three semi-structured focus group interviews. Conventional thematic analysis was employed and results assisted the design of a cross-sectional questionnaire. Sixty-three students completed the questionnaire. ANOVA or chi-square test was used to compare the difference in student’s characteristics, barriers, enablers and perspectives on EY between programme categories. Adjusting student’s characteristics, logistic regressions were conducted to identify the effect of programme categories on the EY experience.

**Results:**

Most students (95% in the questionnaire) agreed that EY was worthwhile and more rewarding than expected. EY was positively regarded for enhancing personal growth and interpersonal relationships. The main barriers were financial difficulties, scholarship issues and insufficient information beforehand. A few students had practical (i.e. accommodation, cultural adaptation) problems. Potential enablers included better financial support, more efficient information exchange and fewer assignments and preparation tasks. Similar barriers were encountered by students across all three categories of EY activities.

**Conclusions:**

Personal growth was the most important benefit of the EY. Barriers were consistent with those identified in the literature except for cultural adaptation, which could be related to Hong Kong’s unique historical context. Financial limitation was the most concerning barrier, as it could result in unequal access to educational opportunities. Better and timely access to scholarships and other funding sources need to be considered.

**Trial registration:**

Ethics approval was obtained from the local Institutional Review Board of The University of Hong Kong/Hospital Authority Hong Kong West Cluster (UW 19-585).

**Supplementary Information:**

The online version contains supplementary material available at 10.1186/s12909-022-03303-z.

## Introduction

Intercalation, or sometimes known as gap year or transition year, is common among undergraduate medicine programs around the world [1–6]. There is a long history of voluntary intercalated enrichment events, in the form of self-directed courses or research projects in between a medical degree from Australia and the United Kingdom [7–10]. They typically take place in between the preclinical and clinical years of study to allow medical students to pursue self-directed learning experience and further personal development in either medical or non-medical fields.

Undertaking an optional intercalated degree showed positive effects on academic performance through the development of deeper learning styles and long-term career outcomes among medical undergraduate students [11]. Similarly, improved learning strategies, increased level of motivation and short-term academic performance were among some of the associated benefits reported by previous studies [12–14]. Aiming to offer similar benefits to its students, the LKS Faculty of Medicine of the University of Hong Kong (HKUMed) introduced a one-year, credit-bearing, compulsory Enrichment Year (EY) in between the preclinical and clinical years of its six-year Bachelor Medicine and Bachelor of Surgery degree programme (MBBS) in 2016.

Considering its credit-bearing nature and incorporation into the medical curriculum, HKUMed’s EY was not designed to simply be a gap year. Instead, it aimed to offer students the means to take charge of their learning and tailor activities to their interests. Students had to choose and plan a local or overseas learning activity under one of the following programme categories: (i) intercalation or exchange programme (IC), (ii) research attachment (RA) or (iii) service and humanitarian work (SH).

Whilst there might be benefits associated with voluntary intercalation, it was unclear whether such benefits could be achieved with a compulsory curriculum [15]. Drawbacks such as cultural impact and financial difficulties affecting students’ experience have been shown to be barriers rather than enablers of the learning experience [15–26]. It was also unclear whether such barriers identified in studies conducted in western countries could be applicable to students from an Asian background. The lack of data on students’ perspective of their experience during intercalation, was the impetus to undertake this study in Hong Kong to explore undergraduate medical students’ perceptions of the EY and to identify the barriers and enablers to optimise the learning experience in different types of EY activities.

## Methods

Medical students in the EY undertook two semester-based or a full-year EY activity that fell under one or any combination of the following three categories:

1. Intercalation or exchange (IC). Students could choose inter- or intra-faculty electives, minor options, or intercalated degrees at the home university or partner institutions, or opt to take courses through the university’s worldwide exchange programme;
2. Research attachment (RA). Students could apply for the 1-year Master of Research in Medicine (MResMed) degree or choose an attachment with principal investigators from Faculty of Medicine in the University of Hong Kong. These attachments began with a 2-week foundational course on basic research skills and ethics, followed by laboratory, clinical or public health research under the supervision of the PI.
3. Service and humanitarian work (SH). Students selected from a roster of local and overseas non-governmental organizations (NGO) who were Faculty partners or self-initiated work with Faculty-approved organizations that engaged in community health promotion, humanitarian relief missions or other service programmes that helped disadvantaged populations. Prior to starting the SH work, all students were required to take a 2-week preparatory course on Global Health that addressed issues such as cultural competency and safety.

As a mandatory, credit-bearing year of study, all students in EY were assessed according to their chosen activity, namely on their final course grades in the IC, and on their academic output and learning attitude for RA and SH. In addition, all students were assessed on a final poster presentation based on their EY as well as experience-sharing in the EY online learning community. Scholarships and financial assistance schemes were available to all students.

### Study design

An exploratory sequential mixed-method design including focus group interviews and subsequently, a cross-sectional questionnaire was used. Students admitted to the MBBS program at HKUMed in 2016/2017 were required to undertake activities to fulfil the EY requirements in 2018/2019, their third year of study. There was a total of 206 medical students (103 female and 103 male) who participated in the EY. Over 90% of students were of Chinese ethnicity. This cohort formed the target population for the study.

Ethics approval was obtained from the local Institutional Review Board of The University of Hong Kong/Hospital Authority Hong Kong West Cluster (UW 19-585).

### Phase I: qualitative stage

Focus group interviews were conducted in September 2019 to explore in-depth student perceptions regarding their learning experience during their EY. Participants were grouped based on their sex, programme duration, programme application methods, category representativeness (i.e. IC, RA or SH), academic outcomes, financial and scholarship status so that students with different characteristics could be represented in each group. To obtain an adequate sample for each category, 3 focus groups of 6 to 7 participants each were recruited to reach saturation of responses, whereby repetitive findings were seen.

Questions on the participants’ views on barriers, enablers and their overall perceptions of the EY were discussed. A preliminary semi-structured interview guide (Supplementary Table 1) was used. Two to four independent facilitators led the interview in Cantonese and/or English. The interviews were audio-taped and transcribed verbatim, with field notes to record non-verbal responses. Summaries were written by two facilitators and compared. A conventional qualitative content analysis method was used to analyse the interview transcripts [27]. Coding categories were inductively derived from the transcripts. The text data was coded independently into main/sub-themes by two investigators manually, with themes marked beside the coded sentences/paragraphs, which were then organized into a tree structure. Inconsistencies in procedure and grouping were resolved by discussion to reach an agreement.

### Phase II: quantitative stage

Questionnaires were conducted between May and June 2020. The questionnaire was designed based on data from the qualitative results and existing literature, in order to test hypotheses generated from qualitative stage and triangulate the whole data set. A hyper-link to a Google form questionnaire was disseminated via email to the entire cohort of MBBS students. The questionnaire collected descriptive demographic information and self-perceived ratings in 4 aspects of the EY experience: perceptions; barriers; enablers and improvements. A 4-point Likert scale (1 = strongly disagree; 2 = disagree; 3 = agree; 4 = strongly agree) was used for a total of 48 questions about views and experiences, while numbers were used for questions related to personal background and academic performance (Additional file 2).

Descriptive statistics were used for student characteristics, barriers, enablers and overall perspective of EY experiences and ANOVA or chi-square tests were used to compare differences. Logistic regressions were conducted to identify the effect of different programme types on barriers, enablers and perspective of EY experience adjusted by student characteristics. Analyses were performed with the latest version of the Stata statistical package. A *p*-value < 0.05 was considered statistically significant.

## Results

### Focus groups findings

#### Views and characteristics

Twenty participants (14 female and 6 male) were invited to participate based on the EY programme type, duration of EY programme and application methods used to enroll in EY programme. A series of sub-themes were identified and grouped into: expectations, enablers, barriers, benefits, suggestions regarding EY (Table 1).

**Table 1:** Qualitative results of the themes on EY experience from students

Sub-themes		Quotes
**Expectations**		
Personal benefits	1.	I was planning to go on an exchange and then travel to different countries to experience different cultures, meet people of different nationalities. (2F, Female, SH)
Academic considerations	2.	I decided to take a whole year minor which was still related to medicine... I worried that if I explored [other] areas for a whole year, I might not be able to pick up medicine again. (2A, Male, IC)
	3.	In the beginning, I did want to get a qualification, so that’s why... I registered for Master of Research in Medicine, but when I didn’t get in, I registered for this [Minor in Kinesiology] one because there was still a qualification. (2A, Male, IC)
Foreseen hinderances	4.	I chose United Kingdom, because... I could use English to communicate. (3B, Male, IC)
**Enablers**		
Administration process enablers	5.	It was helpful for administration, especially for intercalation year because they forged the link and the application was quite straightforward. They already set up the whole thing. (2E, Female, IC [RA])
	6.	In fact, faculty gave us a lot of options, since I couldn’t get into the exchange, I could choose something else... there was still time for me to change. After they approved your plan, you could still change [it] if you could not execute it. (1A, Male, RA & IC)
Financial enablers	7.	I want to thank my family for [financially] supporting me... the school fees [at the overseas schools] were very expensive. (1C, Female, IC & RA)
	8.	Luckily, I was granted some scholarships, and which almost fully funded my project, I didn’t end up paying too much... The biggest scholarship I applied was... sponsored by The Hongkong and Shanghai Banking Corporation. I applied this one around October and November [2017]. After several rounds of interviews, I was informed of the successful result around April [2018]. Therefore... the financial concern was resolved. Indeed, I was kept informed about the results of the other scholarships as well. (3A, Male, RA)
Programme content enablers	9.	My original expectation was that I would be only going to lectures, but the actual content of the course was not like that. There were different teaching methods, [like they] had lots of field trips, for example we went to a physical education college, we got a tour of their college. We got to go to a private gym where you could observe and teach people how do [exercise]. It really had hands on experiences. And there was also a cooking lab... in Causeway Bay... When I first got this offer, I thought this would be quite boring, but after studying for around a month, I started to think that this was quite enjoyable, the content was very interesting, [I was] very satisfied. (2A, Male, IC)
Assistive enablers	10.	I found HRP slightly helpful for my research project, and at least I knew how to approach a lab research or whatever. If I didn’t have the course of health research project, I wouldn’t have known do the Institutional Review Board and ethics approval and etc. Like low key thing is slightly important, if you were doing research in EY. (1E, Female, IC & RA])
	11.	[Laboratory Induction Course] was more focused on molecular and cellular [research], so its focus was on PCR, Western blot etc., so for me it was useful, because that was what I basically did for my research. (1B, Female, RA & IC)
**Barriers**		
Communication barriers	12.	I had already sent emails, private messaged some doctors to see if there were any research opportunities, but there were no replies, and faculty didn’t reply [to the emails], so then I was very frustrated. (2D, Female, RA & IC)
	13.	We were working with the hospitals and schools in Yunnan, I met up with the hospital director and the principal of the school... During our meeting, they were very welcoming and they said they would do their best to help support us… but when the time came for us to carry out our project... none of the front line workers knew who we were and what we were doing, they just thought that we were a group of strange people from Hong Kong... to be honest we didn’t really know if the hospital director supported us, or was if it just a half-hearted response, we just felt that there was no communication between the director and the front line workers, and we didn’t know how to communicate with the front line workers. (2B, Female, IC & SH)
Preparation and application barriers	14.	When I was picking my minor, I did think of [choosing] other courses, however I found out that for some minors, it was impossible to finish it in 1 year, so I sent many emails to ask what courses had to be taken, as some courses need a prerequisite. (1F, Female, IC & SH)
Academic and assessment barriers	15.	I did psychological medicine, a lot of guidelines they teach are the Scottish system, which meant that I cannot apply these back to Hong Kong. So, I’m basically just learnt it for the year... I didn’t take any academic stuff back to Hong Kong... So honestly, I think I was only studying for the purpose of exam not for the purpose of long-term enriching in the academic side. (1E, Female, IC & RA)
	16.	Although my [programme] sounded like it was related to medicine, what I did was actually not relevant to the MBBS curriculum… the only problem is that now that we’re back to the clinical year, it is a bit overwhelming... I feel like I have a lot of things to catch up with after this year commenced and it may be a bit tough... I’m not sure if I’m the only one having this problem, or does everyone else? (3D, Female, IC & RA)
Financial barriers	17.	Some people want to spend a whole year overseas, so they picked intercalation as they could afford it, if you couldn’t afford it, you might take a look at HKU Worldwide, or you would approach the schools yourself, there were many more steps in the process. (2B, Female, IC & SH)
	18.	For some students this was a huge financial burden so they wouldn’t apply to these places [overseas], or they were forced to stay in Hong Kong. [And since they were forced to stay in Hong Kong,] the things they took might not be the things they wanted, they did it just to fulfil the EY requirement, it might not be something that they were interested to do, so for these people they probably thought it wasn’t worth it. (2C, Female, SH & IC)
Personal trait barriers	19.	When we reached there, we were helping out the locals rather than initiating our programmes, because we didn’t know the actual situation, for example we were thinking of organising some tutorial classes... however we didn’t know the children’s educational level and acceptance level, we also didn’t know the arrangement of the local NGO, that’s why the things that we proposed didn’t fit the actual situation, that’s why in the end we assisted the local teaching staff rather than initiating our own activities. At the time I felt like our role was very passive. (2C, Female, SH & IC)
Onsite adaptation barriers	20.	When I went to Yunnan, the locals, like the elderly, spoke their dialect, which resulted in some communication issues. When we distributed questionnaires, some of the elderly were illiterate. But when we read out the questionnaire to them, they still couldn’t understand what we said. (2B, Female, IC & SH)
**Benefits**		
Personal benefits	21	Personally speaking, after experiencing the whole year, I believe this year was needed. If all you do is study, you don’t have time to stop and think, you don’t even know what you’re doing, all you’re doing is studying until you graduate, you haven’t even thought about your life direction, or how you would deal with certain situations, there is no chance for you to mature, so that’s why this year [EY] is very important. (1B, Female, RA & IC)
	22.	I feel like I have become more empathetic and sympathetic. Maybe when we were studying, we were very knowledge-oriented or exam-oriented, but... when we become physicians, we will need to think from the patient’s perspectives. (2C, Female, SH & IC)
	23.	In research, the level of intensity came from the workload but there was no longer the pressure of comparison. In medical school, if you hear other people quiz each other for revision, you may feel like you don’t know anything [you could neither ask nor answer a question like them]. In research, everyone is doing something different, so you can’t see what other people are doing, there is no one to compare with, and so there was no longer that pressure to compare. (1D, Male, IC & RA)
Social benefits	24.	I met a lot of people with different nationalities over there, especially my colleagues in my laboratory who were Turkish, Indian, British, Russian and etc. After chatting with them, I found out that their life experiences and journeys were so distinctive, which gave me a life lesson that I should not limit myself. This was an unexpected benefit. (3A, Male, RA)
	25.	I did get an extra degree within EY and had some experiences which were out of my expectations, such as attending conference, meeting people that I would have never met before, and learning some medically related things. After finishing, I’m grateful that I had an opportunity to do these things before graduation, the only thing is I had a hard time during Year 2. (3D, Female, IC & RA)
Academic benefits	26.	After going to EY, I have a different perspective on [studying in] university: before I thought that studying in university was supposed to be vocationally-oriented or knowledge-based; instead now I think it has to do with self-exploration, you can define what you want to do, then find a way to do what you want to do, the purpose is to learn during the process of achieving a goal rather than to just achieve a goal. I feel like this is a huge change [in thinking]. (1C, Female, IC & RA)
	27.	Like when I was in Year 1 and Year 2, I think I was not very happy, I was more stressed in Year 1 and Year 2... But after staying overseas, I think I have a more stablised mentality, so I feel more comfortable after coming back. Of course, there are still pressures and I’m worried about examinations, but I think I’m calmer and more ready to face challenges after going through the EY. (3A, Male, RA)
Career aspect	28.	I’m very thankful for this year, it helped me realise what I wanted to do. Till now I still don’t know what [sub-]speciality I want to pick, but I think I know what type [area] of specialty is suitable for me. (1C, Female, IC & RA)
	29.	Indeed, some students, who aimed to do research when they were enrolled to [the MBBS programme], might not know what research is like and may not be sure if they want to pursue research [as a career] after graduation. So, I think you can have a chance in EY to experience different types of research... so that you might have a better understanding of what you would like to do in the future. (3D, Female, IC & RA)
	30.	About the service activity in Yunnan... It was like stepping into a mini society, which was very different from our study. We went to an office setting in Yunnan... [It was] very different from when we were studying, there were some conflicts between colleagues, and they would be split into different groups, different sides [office politics], you could hardly imagine that... I feel like this service [attachment] gave me the opportunity to step into society earlier, to see what it would be like to work in society, to know how to get along with others. (2C, Female, SH & IC)
**Suggestions**		
Role of Faculty	31.	I think they could have done another thing to help us, which is planning and preparation. I think the stage we needed most help was when you were planning where to go. (3D, Female, IC & RA)
Administration procedure	32.	I feel like if we have a problem, we need to be able to reach them, like they have to respond to our emails, not wait until a month later before responding to our emails. (2F, Female, SH & SH)
Programme application	33.	I think, if there will continuously be an additional 100 students applying for exchange programme every year, they have to increase the number of quota or coordinate better. (3G, Female, IC)
Programme preparation	34.	I think [they] could reduce the workload from the other aspects, such as HRP, PCP [Patient Care Project], PIP [Professionalism in Practice Programme] and so on. (3B, Male, IC)
	35.	About the scholarships, Faculty of Medicine has published a list of them but up until now I still don’t know if anyone received any of them. To be honest, there was no response of a successful or unsuccessful application after I applied... so I had to apply scholarships not from the Faculty. As I didn’t know when they would announce the results and award the scholarship. (3A, Male, RA)
EY output requirements	36.	Academic output was one of the criteria, I didn’t really want to mention about those things [in the assignment], instead I wanted to talk about the life lessons [that I learnt]. (1D, Male, IC & RA)

*EY* Enrichment year, *IC* Intercalation or exchange programme, *RA* Research attachment, *SH* Service and humanitarian work

#### Expectations

The most frequently mentioned expectations were personal growth, career planning, and opportunities to explore, travel and network (Quote 1). During their EY, students had academic considerations, so they focused on courses that were related to medicine (Quote 2) or aimed to gain an extra qualification (Quote 3). Participants in overseas programmes chose countries where they could communicate to facilitate a smooth experience (Quote 4).

#### Enablers

An easy application process was considered helpful to students (Quote 5). They appreciated the variety of programmes provided by HKUMed, and having sufficient time for executing EY backup plans when required (Quote 6). Two enablers were financial support and scholarship subsidies (Quote 7 and 8). The light workload and the high-quality content of EY programme made their experience more enjoyable (Quote 9). Institutional support, in form of foundation/induction courses in research skills and in global health, gave knowledge and skills to execute their EY projects (Quote 10 and 11). A few participants believed that being highly self-motivated should be the key to their EY.

#### Barriers

Significant barriers were insufficient information exchange between the participants, the university or programme providers (Quote 12 and 13), and the competitiveness of admission requirements (Quote 14). Some participants thought that they were not able to gain any practical knowledge from EY (Quote 15). Furthermore, participants faced bridging difficulties in their studies after EY (Quote 16). Participants felt that financial limitation led to more complicated application processes (Quote 17), as well as unfair access to learning experiences, as some students were compelled to take undesired EY activities (Quote 18). Perceived powerlessness was common for SH activities as participants’ expectation on serving and making a difference were not met (Quote 19). The most common onsite adaptation barriers were communication difficulties and unfamiliar living conditions (Quote 20).

#### Benefits

Most participants were unsure of the benefits of EY beforehand, but after participating, many were supportive as it enhanced their personal growth (Quote 21). A few participants had a higher sense of compassion and empathy after their EY (Quote 22). Others felt less pressure to compare themselves with their peers and focused on their own journeys instead (Quote 23). Enhancing interpersonal networks and broadening horizons were some of the social benefits mentioned (Quote 24 and 25). Some participants praised the EY because it allowed them to have greater ownership of their education (Quote 26), and provided an opportunity to de-stress from the preclinical studies completed before EY (Quote 27). EY also helped some participants to decide on what they wanted to pursue in the future (Quote 28 and 29). Some considered it to be an early workplace experience before graduation, especially for SH and RA programmes (Quote 30).

#### Suggestions

Participants suggested more assistance on planning before the start of EY (Quote 31), as well as timely responses to enquiries would be helpful (Quote 32). A larger exchange quota and more flexible admission requirements were also desirable (Quote 33). Due to the tremendous workload before EY, participants suggested to reduce the workload by shifting the EY activities and/or induction courses to earlier years (Quote 34). Participants believed a more transparent scholarship application process (Quote 35) and more holistic assessment criteria of the learning experiences, including the non-academic output, would be appropriate (Quote 36).

### Questionnaire findings

#### Characteristics of participants

Table 2 showed the characteristics of participants. Sixty-three participants (30.6% of the EY cohort) completed the online questionnaire, including 27 male and 36 female students. The mean and standard deviation of age among the participants was 21.4 and 0.61 years old. Twenty-five students (39.7%) experienced more than one EY programme category. IC was the most popular choice as most of the students (92.1%, *n* = 58) had at least one EY experience in this category. Of these 58 students, 56.9% had solely IC experience whilst 22.4% had IC + RA and 20.7% had IC + SH. Only 38 students (60.3%) received scholarships and 94.7% of these students participated in non-local programmes. Conversely, only 72.0% of the students without a scholarship participated in non-local programmes during their EY. Also, only 55% of scholarship holders did not have a multi-category EY experience, compared with 84% of the non-scholarship holders.

**Table 2:** Characteristics of participants

	Overall (*N* = 63)
Age, years	21.4 (0.61)
Male	27 (42.9%)
Any scholarship	38 (60.3%)
Any Non-local program	54 (85.7%)
All faculty-coordinated module	27 (42.9%)
Any self-initiated module	36 (57.1%)
Any multi-category experience	25 (39.7%)
Enrolled intercalated degree, exchange, minor program	58 (92.1%)
Enrolled research attachment	15 (23.8%)
Enrolled service/humanitarian work	15 (23.8%)

#### Perceptions

**Fig. 1:**
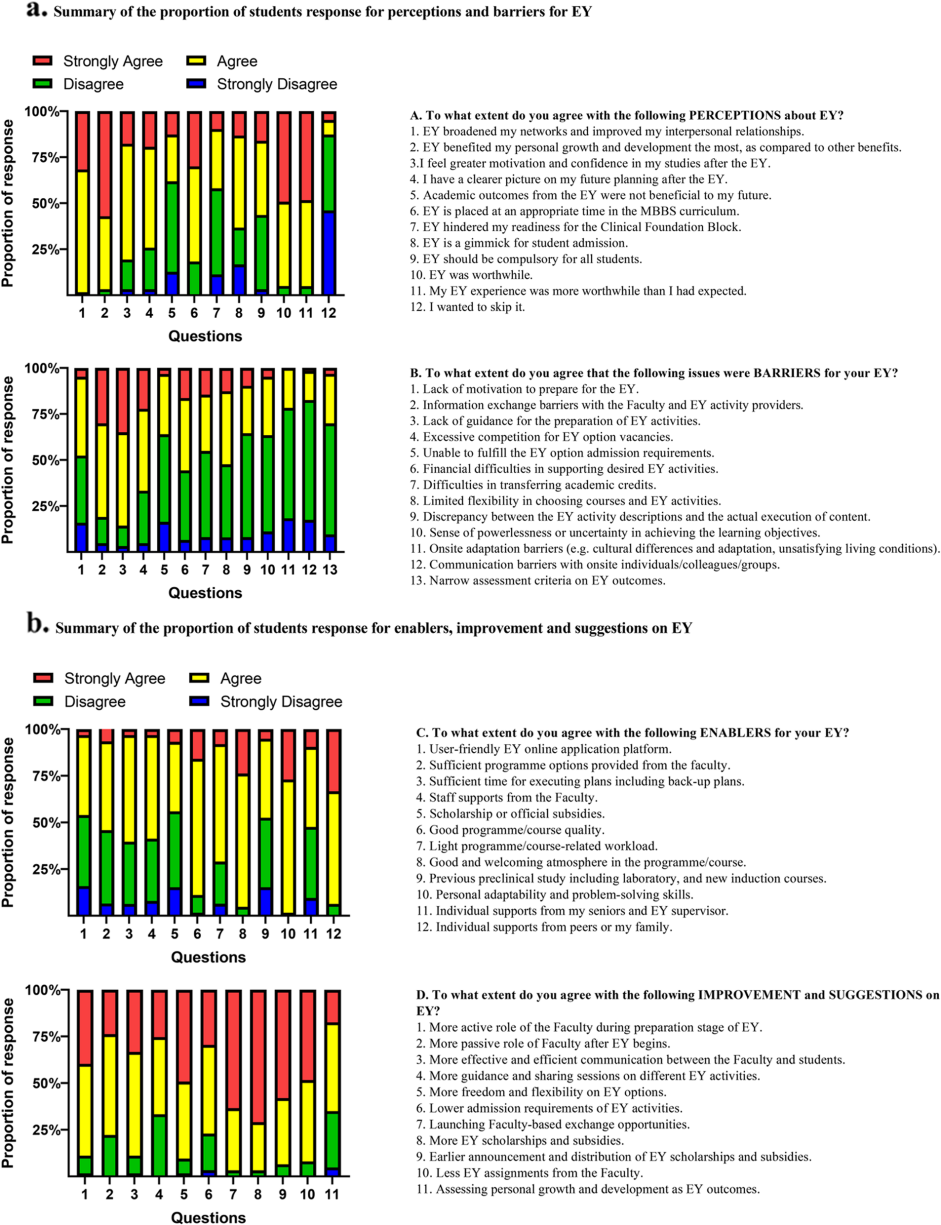
**a** Summary of the proportion of students response for perceptions and barriers for EY. **b** Summary of the proportion of students response for enablers, improvement and suggestions on EY

EY was well perceived especially on personal growth and interpersonal relationship. Nearly all students (95%) agreed that EY was more worthwhile than they had expected. 83% of the participants agreed that “EY was placed at an appropriate time in the MBBS curriculum”. Student generally shared similar perceptions about EY, irrespective of their EY categories or scholarship status. A significantly higher proportion of students with non-local EY agreed that “their EY had broadened their network and improved their interpersonal skills” and “their EY experience was more worthwhile than I had expected”. Further, students who had scholarships were highly likely to agree on “EY should be compulsory” (OR: 5.19 (CI: 1.18,22.82, *p* = 0.029), as well as for students who had self-initiated EY activities (OR: 3.20 (CI: 1.03, 9.89, *p* = 0.044)) (Fig. 1a, Supplementary Fig. 1, and Supplementary Table 2a and b).

#### Barriers

Most students agreed on “the lack of guidance on preparation of EY activities”, “information exchange barriers between university and EY providers”, and “excessive competition for EY option vacancies”. Conversely, less than 25% students agreed that practical adaptation (such as accommodations, cultural adaptation) and onsite communication were barriers. Students who participated in SH were unlikely to agree on “lack of motivation to prepare for EY” (OR: 0.12 (CI: 0.02,0.79, *p* = 0.027)), and “unable to fulfill the EY option admission requirement” (OR: 0.01 (CI: 0.001, 0.14, *p* < 0.001)). Students who did not have any EY scholarships had a significantly higher agreement rate on “unable to fulfil admission requirement”. Students with only local EY had a higher rate on agreeing that admission requirement was one of their barriers. Students with multi-category EY agreed more with financial difficulties in supporting EY activities, and highly correlated to having financial difficulties in supporting their desired options (OR: 6.60 (CI: 1.24, 35.22, *p* = .027)) (Fig. 1a, Supplementary Fig. 2, and Supplementary Table 2a and b).

#### Enablers

Students had a high agreement rate on “good quality of the programme”, “welcoming atmosphere of the EY programme”, “personal problem-solving skills” and “individual supports from peers or family”. Comparatively, only half of the student agreed that support from senior students or EY supervisors were their enabler. Students who had participated in SH had a higher agreement rate on “light workload” as their enablers, and also were significantly more likely to agree to such statement (OR: 12.56 (CI: 1.06,149.44, *p* = .045). As expected, scholarship holders were more likely to agree to “having scholarship” as an enabler (OR: 7.86 (CI: 1.27,48.48, *p* = .026)) (Fig. 1b, Supplementary Fig. 3, and Supplementary Table 2a and b).

#### Improvements and suggestions

All statements on “improvement and suggestions” had an agreement rate of 50% or above (“Agree” or “Strongly Agree”). “Launching more faculty-based exchange opportunities” and “more EY scholarship and subsidies” were statements that had the highest frequency of agreement. The statement with the lowest agreement was on “assessing personal growth and development as EY outcomes”. This indicated that students after EY generally were able to observe their own development and understand the value of EY. Students with different types of EY did not show a significant difference in their responses. The only difference observed was between students with only one and multi-category EY experience, with more students in the latter agreeing that communication between university and students needed to be improved (Fig. 1b, Supplementary Fig. 4, and Supplementary Table 2a and b).

## Discussion

From both analyses, non-local EY programmes were better perceived. A university-linked programme list and scholarships were two of the most mentioned enablers in our mixed-method study. Generally, our results on barriers to EY aligned with existing evidence, with only culture adaptation not seen as significant by our medical students. Better administrative support was one of the major suggestions for improving EY.

Differences were observed on academic expectations regarding earning extra qualifications: some aimed to gain additional qualifications through EY for career advancement while others treated the qualifications as a bonus. Some participants admitted they had concerns about the academic value of their EY activities towards their medical study. However, as EY was designed to enhance the total learning experiences of students that deliberately discouraged medical-related activities [28], their experiences actually improved the mental state of some participants allowing them to de-stress from the preclinical studies completed before EY. They reflected on the meaning of studying at a university and could focus on their own journeys rather than comparing themselves with others, suggesting that students became more confident and motivated to confront the new school year and its new challenges. Quantitative results showed that EY was not expected to be a worthwhile component in the MBBS curriculum, but after EY, students found that they perceived EY differently, and it had become something that they would not like to skip in hindsight.

The most significant enabler identified by the qualitative analyses was the university-linked programme list. Participants found the preparation and application procedures to be the most time-consuming part of the EY. Thus, the list provided by the university reduced the work associated with self-initiated EY activities. However, quantitative analyses indicated that the most apparent enabler was scholarships. Students with scholarships might have higher academic achievements originally, which could explain their lower agreement rate on “unable to fulfill admission requirements” to more competitive EY options. This further correlated to students’ perception of EY significantly. Our results also suggested that students with SH experience were more likely to have a lighter workload and were unlikely to lack motivation. This suggests that the different types of EY could be an enabler for students depending on the workload.

Our results on the “barriers of EY” were mostly consistent with the studies conducted on the voluntary-based intercalating activities [15–26]. Financial constraint was the most significant reason causing differences in satisfaction level as participants tended to believe that they would be able to have an EY with simpler planning and more choices if they were not limited by financial constraints. Thus, financial disparity created a sense of unfairness in accessing desired learning experiences. University students value the importance of a fair opportunity in education believing that both rich and poor students should receive equal opportunities [29]. Students with financial limitations were less motivated to use the opportunities provided by EY to enrich themselves.

Interestingly, cultural adaptation, onsite coordination and the execution of EY activities by the onsite individuals or groups were, in general, not perceived as barriers. The uniqueness of Hong Kong’s historic background could potentially explain why problems with cultural adaptation was not observed among our students compared to those in the literature. Hong Kong, which is currently under the sovereignty of China, had been a colony of the United Kingdom. Local Hong Kong students, being at least bilingual (fluent in English and Cantonese), had expressed their sense of “dual identity” and pride at Hong Kong being “a place where East meets West” [30]. Growing up in such environment, it is unsurprising that cultural adaptation was not a notable barrier.

Participants wished to have more overseas opportunities with less financial difficulties, and less hinderances, which included insufficient information exchange and online assignments. Suggestions from both results on EY improvement focused on the administrative support, such as “the university playing a more active role in assisting the preparation procedures”, “giving timely announcement of information and response from inquiries”, “providing more scholarships with earlier distribution of subsidies” and “timely review of the online tasks”.

Our study had three major limitations. Firstly, the focus group interview findings were based on self-reported recollections by the participants but the recall bias should be small as the questions asked about personal attitudes and opinions only. Secondly, participant recruitment was on voluntary basis. Therefore, less-motivated participants who might have more negative comments on EY might not be recruited to participate in the focus group interviews. For the questionnaire, one major limitation was the small sample size. Only 30.6% of the EY cohort participated in the questionnaire, and thus potential selection bias might affect the findings and variances within subgroup might not be detected by the analysis.

## Conclusion

From the students’ perspective, apart from cultural adaptation, most of our findings on barriers perceived were similar to those that were reported in studies from western countries. Hong Kong students faced similar barriers regardless of the types of EY that they had. Financial limitation was the most concerning barrier, as it could result in unequal access to educational opportunities. The most obvious benefits were perceived personal growth and development, and that EY nurtured a more mature cohort of medical students.

## Supplementary Information

**Additional file 1: Supplementary Table 1.** Semi-structured interview guide. **Supplementary Table 2.** Regression analysis in the perceptions, barriers, enables, improvements and suggestions about EY by subgroups. **Supplementary Figure 1.** Summary of the proportion of students response for perceptions about EY by subgroups. **Supplementary Figure 2.** Summary of the proportion of students response for barriers for EY by subgroups. **Supplementary Figure 3.** Summary of the proportion of students response for enablers for EY by subgroups. **Supplementary Figure 4.** Summary of the proportion of students response for improvement and suggestions on EY by subgroups.

**Additional file 2.** Questionnaire.

## Data Availability

The datasets used and/or analyzed during the current study are available from the corresponding author on reasonable request.
