# Supplementary material for: Identifying and articulating the student experience in the Intercalated Enrichment Year
Source: BMC Med Educ. 2022 Apr 4;22:246. doi: 10.1186/s12909-022-03303-z (PMC8981939; doi:10.1186/s12909-022-03303-z)

**Supplementary table 1. Semi-structured interview guide**

| Introduction: explain purpose of study and focus group.  “We are meeting with you today to better understand medical students’ learning experience in term of barriers, facilitators and interpretation across *Intercalation or Exchange, Research Attachment, Service and Humanitarian Work* during enrichment year to inform student support needs and optimize learning experiences. In this discussion, we are most interested in your perceptions and decisions you have made about enrichment year. All you have shared during this interview will be kept anonymous and confidential.”   1. How and why did you choose your *EY* activity? What factors did you consider when choosing them? Did you encounter any barrier or have any difficulty in choosing your activity? If so, how did you overcome them? 2. What barriers did you encounter during your enrichment year? How?  How did you, or other parties or individuals help you, overcome these things/factors? Was there anything/facilitators smooth your *EY* experience? 3. How did your learning experiences match with your expectations of enrichment year?  How would you think you are benefited from your *EY* activity? 4. What do you think about the role of Faculty of Medicine in supporting students for enrichment year? If your EY activities were Faculty provided, how do you think of the quality of activity?  What could be done to make the experience better and who could make this improvement? 5. What is your interpretation about the goals of Faculty of launching Enrichment Year programme in the 130 Curriculum before and after your learning experiences?   Do these goals match with your own expectations on Enrichment Year? How? Do you think enrichment year should be compulsory? Why?   1. What would be your overall comment on enrichment year?   Conclusion: students should be thanked. |
| --- |


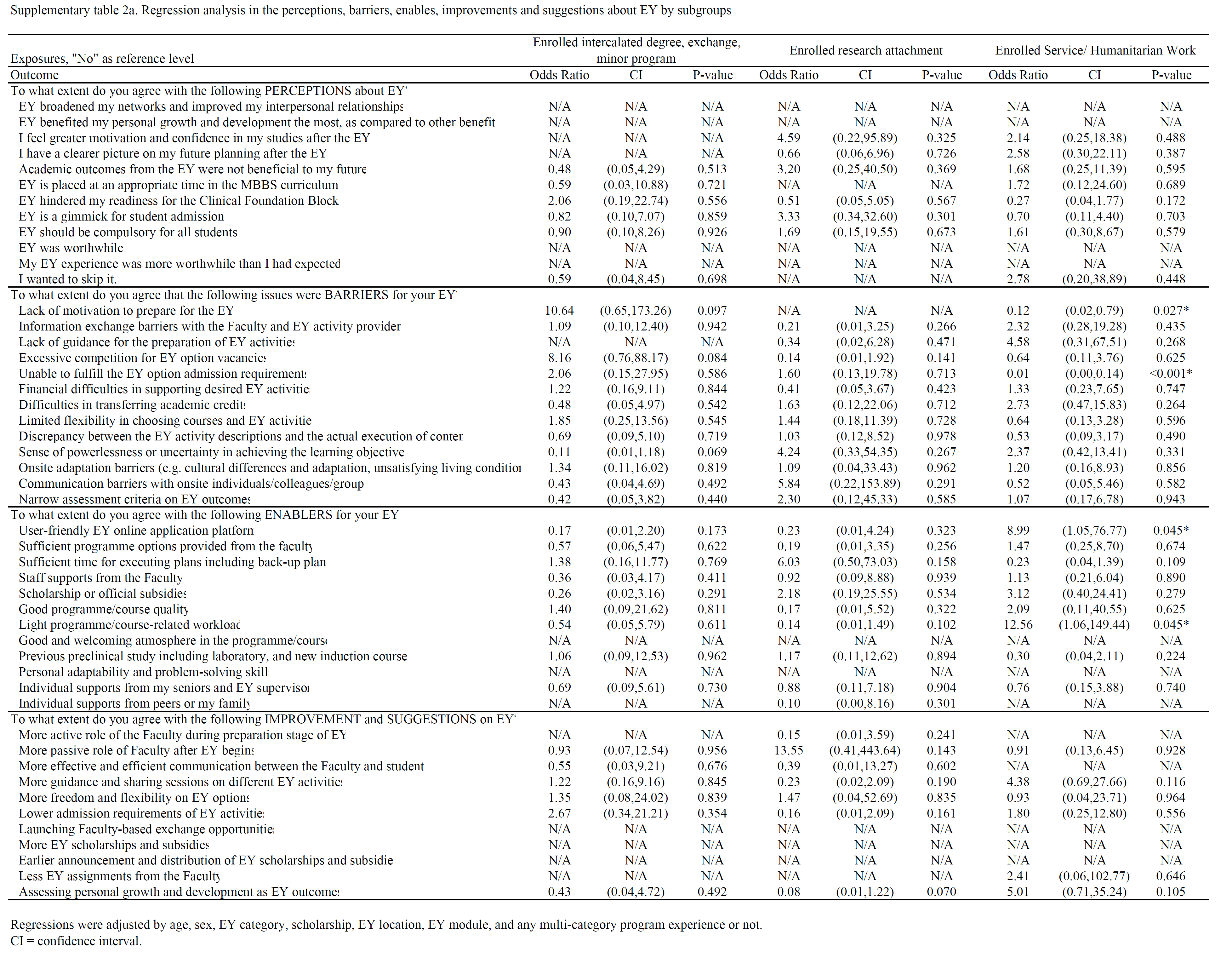


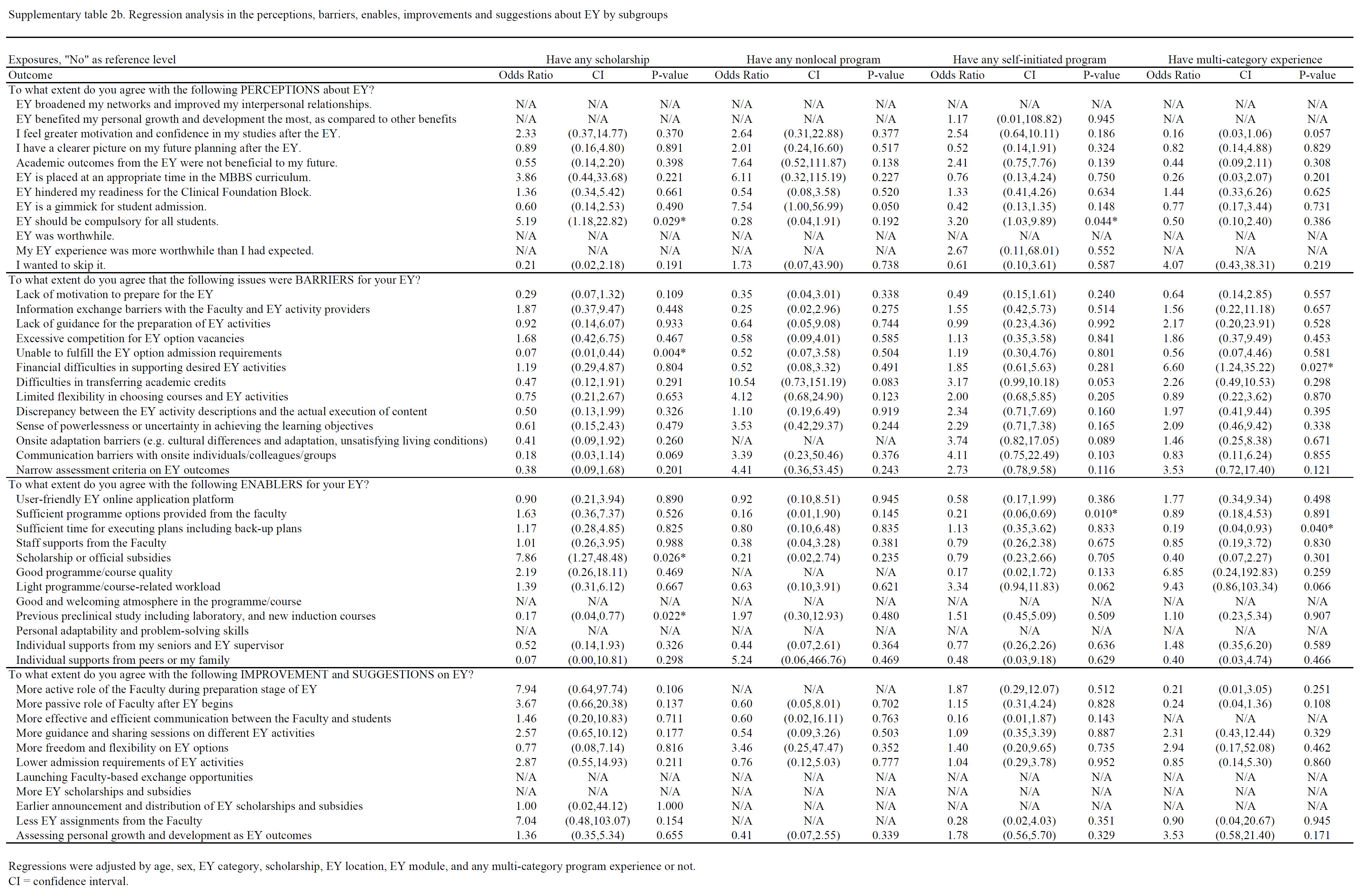


Supplementary figure 1 Summary of the proportion of students response for perceptions about EY by subgroups


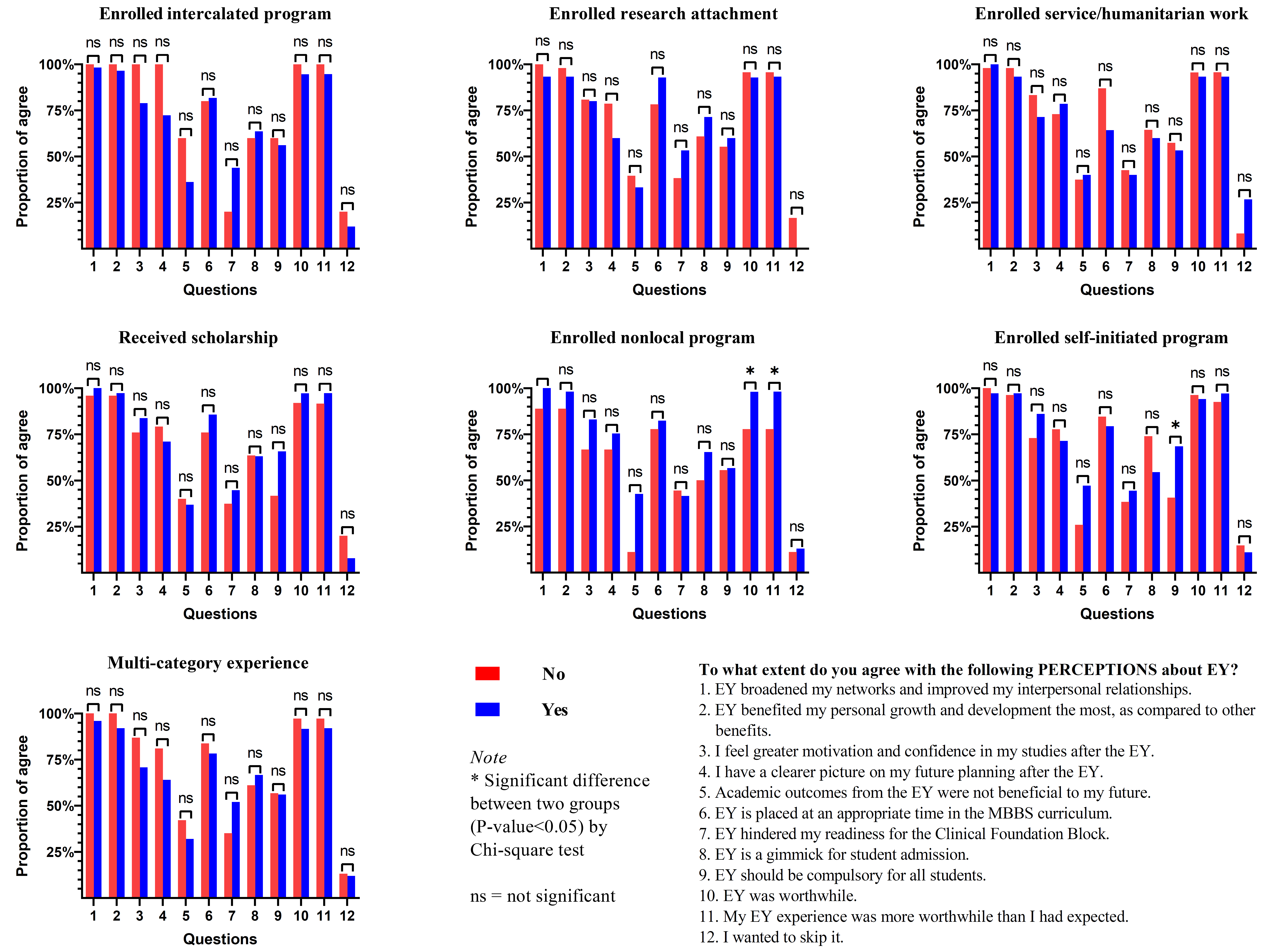


Supplementary figure 2 Summary of the proportion of students response for barriers for EY by subgroups


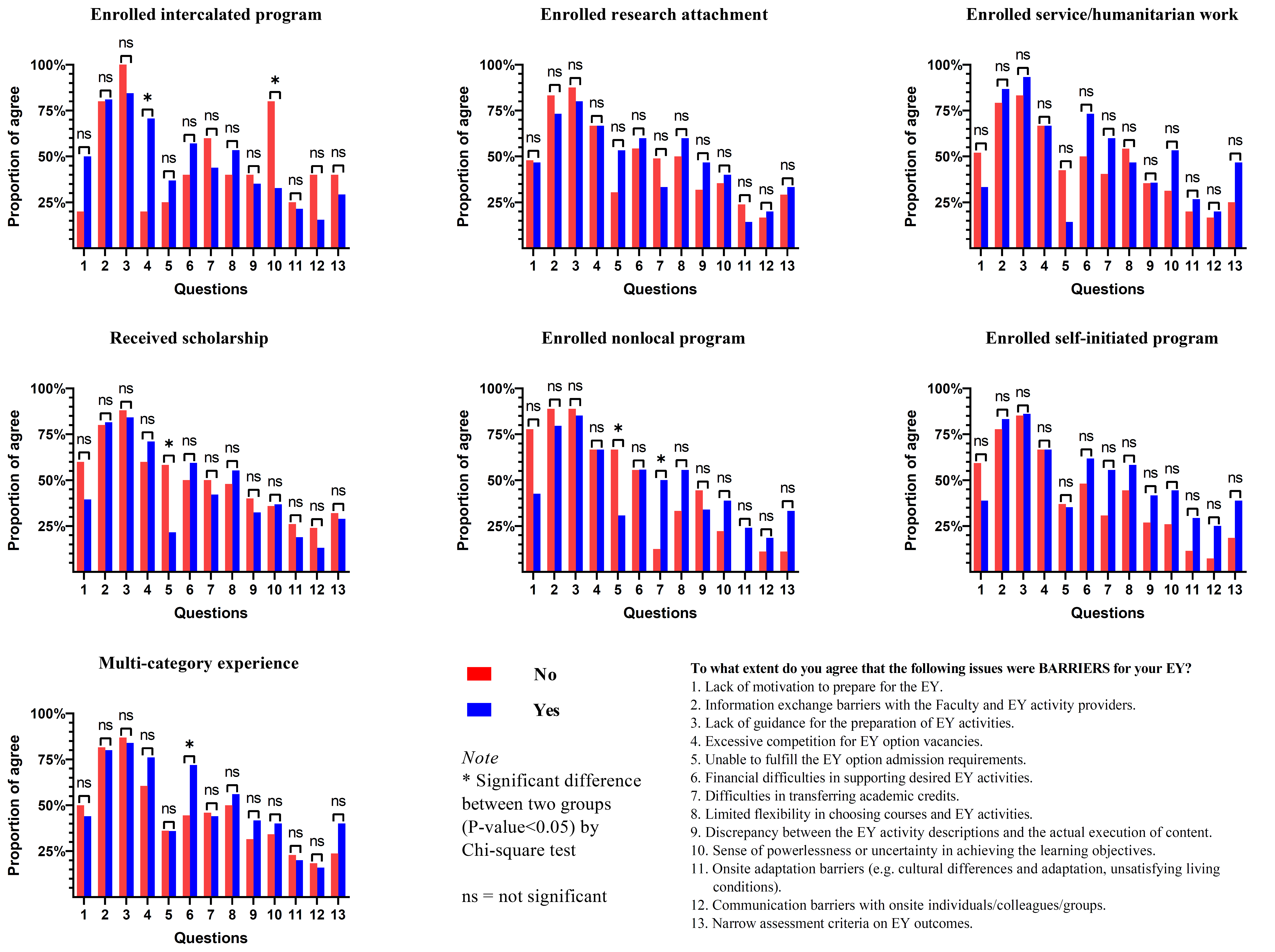


Supplementary figure 3 Summary of the proportion of students response for enablers for EY by subgroups.


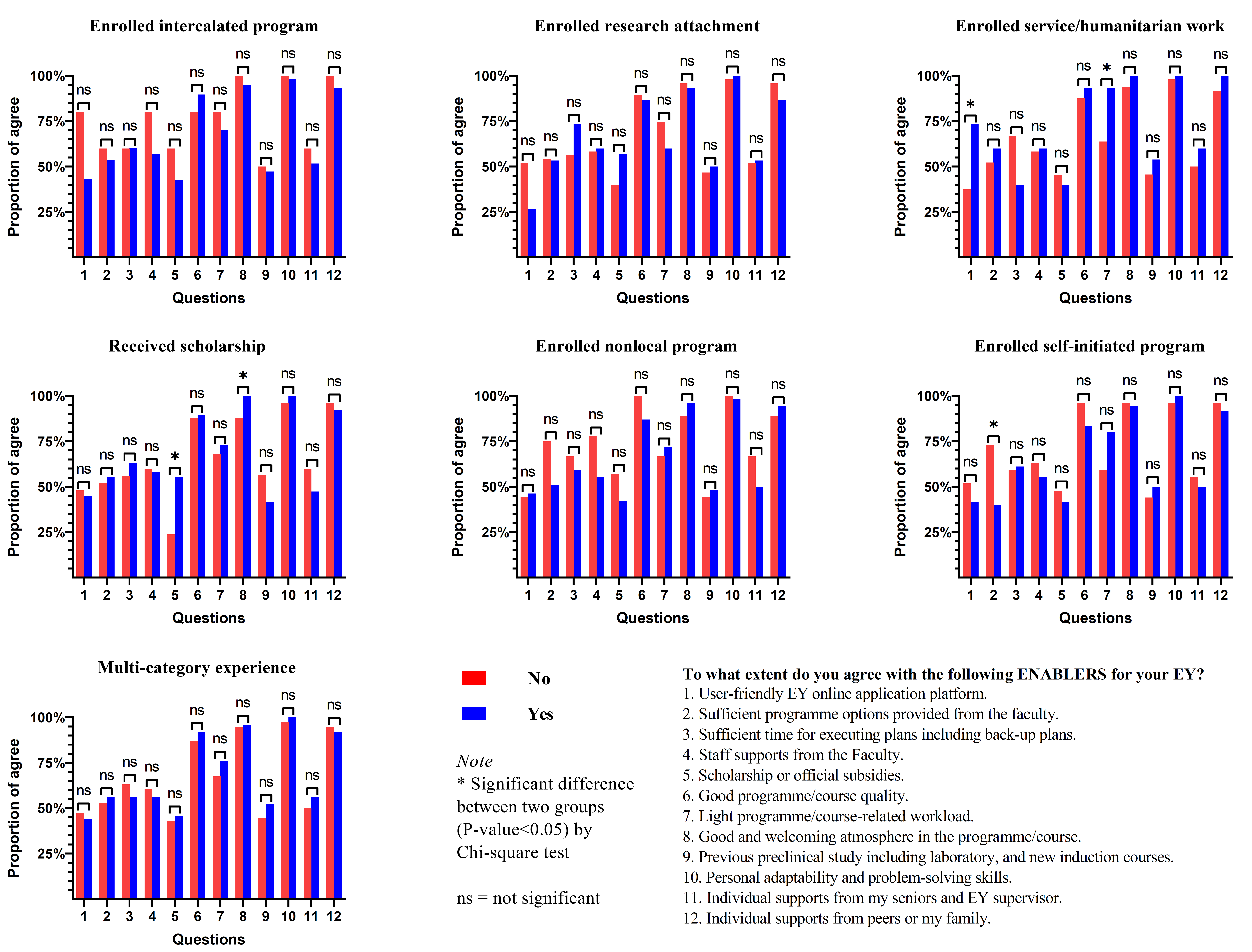


Supplementary figure 4 Summary of the proportion of students response for improvement and suggestions on EY by subgroups


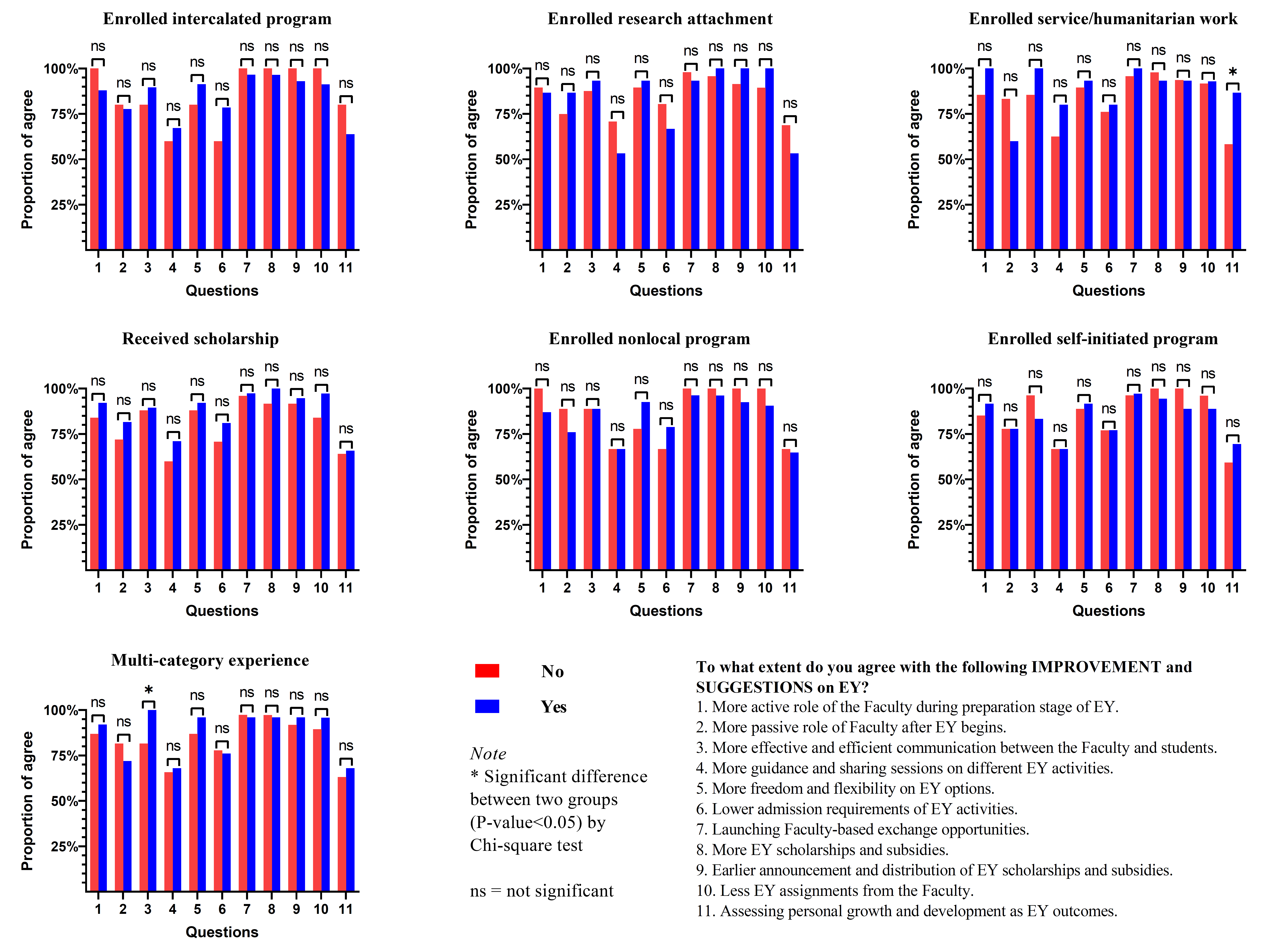

Supplement: Supplementary file 1 — Additional file 1: Supplementary Table 1. Semi-structured interview guide. Supplementary Table 2. Regression analysis in the perceptions, barriers, enables, improvements and suggestions about EY by subgroups. Supplementary Figure 1. Summary of the proportion of students response for perceptions about EY by subgroups. Supplementary Figure 2. Summary of the proportion of students response for barriers for EY by subgroups. Supplementary Figure 3. Summary of the proportion of students response for enablers for EY by subgroups. Supplementary Figure 4. Summary of the proportion of students response for improvement and suggestions on EY by subgroups. [file 12909_2022_3303_MOESM1_ESM.docx]
