# Supplementary material for: Identifying and articulating the student experience in the Intercalated Enrichment Year
Source: BMC Med Educ. 2022 Apr 4;22:246. doi: 10.1186/s12909-022-03303-z (PMC8981939; doi:10.1186/s12909-022-03303-z)
Supplement: Supplementary file 2 — Additional file 2. Questionnaire. [file 12909_2022_3303_MOESM2_ESM.docx]

**Additional file 2. Questionnaire**

**Identifying and articulating the student learning experience in the MBBS 130 curriculum Enrichment Year**

**Information Collection and Participation Consent Statement**

**Introduction and Procedure**

In 2016/2017, Li Ka Shing Faculty of Medicine implemented the Bachelor of Medicine and Bachelor of Surgery Programme (MBBS) 130 curriculum. Students were given opportunities to explore their areas of interest in either medical or non-medical fields during their Enrichment Year (EY). The goal is to enhance students overall learning experience and further personal development. We are interested in the views and learning experience of medical students in the MBBS 130 curriculum EY. The study results will be helpful to refine and develop the student support mechanisms and resources in the EY of the curriculum.

Medical students of the University enrolled in 2016-17 academic year (the first batch of students in 130 curriculum) will be recruited to complete the questionnaire. The questionnaire will take approximately 15 minutes to complete, covering questions regarding your views and learning experience in the MBBS 130 curriculum EY.

**Potential Benefits**

We expect that participation in this study will provide you the opportunity to provide us insights on the future development and improvement of the curriculum by identifying the barriers and facilitators to achieving the proposed learning outcomes in the EY. Information learnt regarding the barriers and facilitators to research attachments can also provide guidance to the University on how to promote and support undergraduates in research. Besides, information on the barriers and facilitators to international exchanges, and service and humanitarian work can help to inform the HKU Horizons “Learning Beyond Hong Kong” initiative.

**Participation and Withdrawal**

Your participation is completely voluntary. You may refuse to participate or withdraw at any time you wish. You may choose whether or not to answer any specific question.

**Confidentiality**

All the information obtained is for research purpose only and will be kept strictly confidential. Under the laws of the Hong Kong Special Administrative Region and, in particular, the Personal Data (Privacy) Ordinance, Cap 486, you enjoy or may enjoy rights for the protection of the confidentiality of your personal data, such as those regarding the collection, custody, retention, management, control, use (including analysis or comparison), transfer in or out of Hong Kong, non-disclosure, erasure and/or in any way dealing with or disposing of any of your personal data in or for this study.

**Study Review**

This study is reviewed and approved by the Institutional Review Board of the University of Hong Kong / Hospital Authority Hong Kong West Cluster.

**Questions and Concerns**

If you have any questions about the study, please feel free to contact the principal investigator Dr. Yuk Fai WAN via email (yfwan@hku.hk). Thank you very much.

By signing and dating this consent form, you agree to allow the collection, custody, retention, management, control, and use your personal data in this study in ways described above. For any query, you should consult the Privacy Commissioner for Personal Data or his office (Tel No. 2827 2827 ) as to the proper monitoring or supervision of your personal data protection so that your full awareness and understanding of the significance of compliance with the law governing privacy data is assured.

**Student Informed Consent Form**

The following statements are to check that you understand and consent to the procedures of this research:

1. I confirm that I have understood (or had someone explained) the information for this study. I have the opportunity to ask questions about the project and I understand why the research is being done and any risks involved.
2. I understand that my participation is voluntary.
3. I agree to take part in the study.
4. I understand that all information that I provide to the research team will be kept confidential and only the research team will see it.
5. I understand how the data will be collected, that giving data for this research is voluntary and that I am free to withdraw my approval for use of the sample at any time, without giving reason and without my legal rights being affected.
6. I understand that I am free to withdraw from the study at any time, without giving reason or any legal rights being affected.
7. By signing a written informed consent form, you are authorizing the Research Ethics Committee (REC) and Institutional Review Board of the University of Hong Kong / Hospital Authority Hong Kong West Cluster (IRB) will be granted direct access to the participant’s study data for data verification.
8. I will get a copy of this consent form for record.

*Please tick the most appropriate box for the following items.*

| **Q1. To what extent do you agree with the following *PERCEPTIONS* about EY?** | | Strongly disagree | Disagree | Agree | Strongly agree | Not applicable |
| --- | --- | --- | --- | --- | --- | --- |
|  |  | 1 | 2 | 3 | 4 | 5 |
|  | EY broadened my networks and improved my interpersonal relationships. | □ | □ | □ | □ | □ |
|  | Compared to other benefits, EY benefited my personal growth and development the most. | □ | □ | □ | □ | □ |
|  | I feel greater motivation and confidence in my studies after the EY. | □ | □ | □ | □ | □ |
|  | I have a clearer picture on my future planning after the EY. | □ | □ | □ | □ | □ |
|  | Academic outcomes from the EY were not beneficial to my future. | □ | □ | □ | □ | □ |
|  | EY is placed at an appropriate time in the MBBS curriculum | □ | □ | □ | □ | □ |
|  | EY hindered my readiness for the Clinical Foundation Block. | □ | □ | □ | □ | □ |
|  | EY is a gimmick for student admission. | □ | □ | □ | □ | □ |
|  | EY should be compulsory for all students. | □ | □ | □ | □ | □ |
|  | EY was worthwhile | □ | □ | □ | □ | □ |
|  | My EY experience was more worthwhile than I had expected. | □ | □ | □ | □ | □ |
|  | I wanted to skip it | □ | □ | □ | □ | □ |

| **Q2.To what extent do you agree that the following issues were *BARRIERS* for your EY?** | | Strongly disagree | Disagree | | Agree | | Strongly agree | | Not  applicable | |
| --- | --- | --- | --- | --- | --- | --- | --- | --- | --- | --- |
|  |  | 1 | 2 | | 3 | | 4 | | 5 | |
| a. | Lack of motivation to prepare for the EY. | □ | □ | | □ | | □ | | □ | |
| b. | Information exchange barriers with the Faculty and EY activity providers | □ | □ | | □ | | □ | | □ | |
| c. | Lack of guidance for the preparation of EY activities | □ | □ | | □ | | □ | | □ | |
| d. | Excessive competition for EY option vacancies | □ | □ | | □ | | □ | | □ | |
| e. | Unable to fulfill the EY option admission requirements | □ | □ | | □ | | □ | | □ | |
| f. | Financial difficulties in supporting desired EY activities | □ | | □ | | □ | | □ | | □ |
| g. | Difficulties in transferring academic credits | □ | | □ | | □ | | □ | | □ |
| h. | Limited flexibility in choosing courses and EY activities | □ | | □ | | □ | | □ | | □ |
| i. | Discrepancy between the EY activity descriptions and the actual execution of content | □ | | □ | | □ | | □ | | □ |
| j. | Sense of powerlessness or uncertainty in achieving the learning objectives | □ | | □ | | □ | | □ | | □ |
| k. | Onsite adaptation barriers (e.g. cultural differences and adaptation, unsatisfying living conditions) | □ | | □ | | □ | | □ | | □ |
| l. | Communication barriers with onsite individuals/colleagues/groups | □ | | □ | | □ | | □ | | □ |
| m. | Narrow assessment criteria on EY outcomes | □ | | □ | | □ | | □ | | □ |
|  |  |  | |  | |  | |  | |  |

| **Q3. To what extent do you agree with the following *ENABLERS* for your EY?** | | Strongly disagree | Disagree | Agree | Strongly agree | Not applicable |
| --- | --- | --- | --- | --- | --- | --- |
|  |  | 1 | 2 | 3 | 4 | 5 |
| a. | User-friendly EY online application platform | □ | □ | □ | □ | □ |
| b. | Sufficient programme options provided from the faculty | □ | □ | □ | □ | □ |
| c. | Sufficient time for executing plans including back-up plans | □ | □ | □ | □ | □ |
| d. | Staff supports from the Faculty | □ | □ | □ | □ | □ |
| e. | Scholarship or official subsidies | □ | □ | □ | □ | □ |
| f. | Good programme/course quality | □ | □ | □ | □ | □ |
| g. | Light programme/course-related workload | □ | □ | □ | □ | □ |
| h. | Good and welcoming atmosphere in the programme/course | □ | □ | □ | □ | □ |
| i. | Previous preclinical study including laboratory, and new induction courses | □ | □ | □ | □ | □ |
| j. | Personal adaptability and problem-solving skills | □ | □ | □ | □ | □ |
| k. | Individual supports from my seniors and EY supervisor | □ | □ | □ | □ | □ |
| l. | Individual supports from peers or my family | □ | □ | □ | □ | □ |

| **Q4. To what extent do you agree with the following *IMPROVEMENT and SUGGESTIONS* on EY?** | | Strongly disagree | Disagree | Agree | Strongly agree | Not applicable |
| --- | --- | --- | --- | --- | --- | --- |
|  |  | 1 | 2 | 3 | 4 | 5 |
| a. | More active role of the Faculty during preparation stage of EY | □ | □ | □ | □ | □ |
| b. | More passive role of Faculty after EY begins | □ | □ | □ | □ | □ |
| c. | More effective and efficient communication between the Faculty and students | □ | □ | □ | □ | □ |
| d. | More guidance and sharing sessions on different EY activities | □ | □ | □ | □ | □ |
| e. | More freedom and flexibility on EY options | □ | □ | □ | □ | □ |
| f. | Lower admission requirements of EY activities | □ | □ | □ | □ | □ |
| g. | Launching Faculty-based exchange opportunities | □ | □ | □ | □ | □ |
| i. | More EY scholarships and subsidies | □ | □ | □ | □ | □ |
| j. | Earlier announcement and distribution of EY scholarships and subsidies | □ | □ | □ | □ | □ |
| k. | Less EY assignments from the Faculty | □ | □ | □ | □ | □ |
| l. | Assessing personal growth and development as EY outcomes | □ | □ | □ | □ | □ |

**5) Background**

1. Age: _____________
2. Gender: □ Male □ Female

1. EY Category

*Please tick the appropriate answer.*

|  | Intercalated degree | HKUWW Exchange | Minor/ Electives | Research Attachment | Service/ Humanitarian Work |
| --- | --- | --- | --- | --- | --- |
| Semester 1 (For sem-based module) |  |  |  |  |  |
| Semester 2 (For sem-based module) |  |  |  |  |  |
| Full year |  |  |  |  |  |

1. EY Module

*Please tick the appropriate answer.*

|  | Self-initiated Module | Faculty-coordinated Module |
| --- | --- | --- |
| Semester 1 (For sem-based module) |  |  |
| Semester 2 (For sem-based module) |  |  |
| Full year |  |  |

| 1. EY Location   *Please tick the appropriate box for each of your EY activity* | Hong Kong | China | Asia including Taiwan (Other than China) | Outside Asia |
| --- | --- | --- | --- | --- |
| a. Semester 1 (For sem-based module) | □ | □ | □ | □ |
| b. Semester 2 (For sem-based module) | □ | □ | □ | □ |
| c. Full year | □ | □ | □ | □ |

1. Scholarship

*Please specify the* ***TOTAL AMOUNT*** *for each of your EY activity.*

| Semester 1 (For sem-based module) |  |  |
| --- | --- | --- |
| Semester 2 (For sem-based module) |  |  |
| Full year |  |  |
| Unclassified scholarship |  |  |
